# Large-Scale Molecular Dynamics of Anion-Exchange Membranes: Molecular Structure of QPAF-4 and Water Transport

**DOI:** 10.3390/membranes15090266

**Published:** 2025-09-02

**Authors:** Tetsuro Nagai, Takumi Kawaida, Koji Yoshida

**Affiliations:** 1 Department of Chemistry, Faculty of Science, Fukuoka University, 8-19-1 Nanakuma, Jonan-ku, Fukuoka 814-0180, Japan; kyoshida@fukuoka-u.ac.jp; 2 Graduate School of Science, Fukuoka University, 8-19-1 Nanakuma, Jonan-ku, Fukuoka 814-0180, Japan

**Keywords:** anion-exchange membranes (AEM), molecular dynamics simulations, structure factor, pair distribution function, water transport

## Abstract

Understanding the molecular structure and water transport behavior in anion-exchange membranes (AEMs) is essential for advancing efficient and cost-effective alkaline fuel cells. In this study, large-scale all-atom molecular dynamics simulations of QPAF-4, a promising AEM material, were performed at multiple water uptakes (*λ* = 2, 3, 6, and 13). The simulated systems comprised approximately 1.4 to 2.1 million atoms and spanned approximately 26 nm, thus enabling direct comparison with both wide-angle X-ray scattering (WAXS) and small-angle X-ray scattering (SAXS) experiments. The simulations successfully reproduced experimentally observed structure factors, accurately capturing microphase-separated morphologies at the mesoscale (~8 nm). Decomposition of the SAXS profile into atom pairs suggests that increasing water uptake may facilitate the aggregation of fluorinated alkyl chains. Furthermore, the calculated pair distribution functions showed excellent agreement with WAXS data, suggesting that the atomistic details were accurately reproduced. The water dynamics exhibited strong dependence on hydration level: At low water uptake, mean squared displacement showed persistent subdiffusive behavior even at long timescales (~200 ns), whereas almost normal diffusion was observed when water uptake was high. These results suggest that water mobility may be significantly influenced by nanoconfinement and strong interactions exerted by polymer chains and counterions under dry conditions. These findings provide a basis for the rational design and optimization of high-performance membrane materials.

## 1. Introduction

Polymer electrolyte fuel cells (PEFCs) have attracted significant attention as a clean energy conversion device that operates efficiently and emits only water as a byproduct [1,2]. The performance of PEFCs is significantly influenced by the properties of the ion-exchange membranes utilized. When proton-exchange membranes (PEMs) are employed, the system is referred to as a proton-exchange membrane fuel cell (PEMFC). The commercialization of PEMFCs has occurred in automotive and residential applications. A notable disadvantage of these systems is the reliance on expensive acid-resistant platinum as an electrocatalyst.

Anion-exchange membrane fuel cells (AEMFCs) are attracting increasing attention as a cost-effective alternative to PEMFCs [3,4,5,6,7,8]. This is due to the alkaline environments within the AEMFCs that enable the use of cost-effective platinum-free catalysts, thereby reducing overall costs. Furthermore, the alkaline conditions can enhance the oxygen reduction reaction rate. However, AEMFCs have not been extensively adopted due to their current limitations in performance and durability. A salient issue pertaining to the performance is the management of water transport [9,10]. In AEMFCs, the generation and consumption of water molecules occur on anodes and cathodes, respectively. To maintain the proper hydration of both electrodes and AEM, the transport of water molecules must be meticulously regulated.

**Figure 1:**
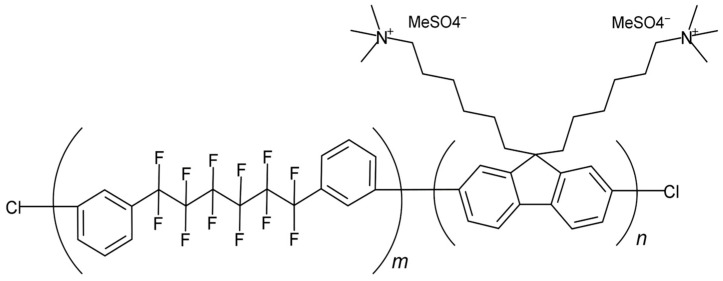
Chemical structure of QPAF-4.

In this study, we focused on a promising AEM material, QPAF-4 [11,12,13,14,15,16,17,18] (chemical structure is shown in Figure 1). The QPAF-4 polymer consists of hydrophobic residues (hereafter referred to as PAF) containing fluorinated alkyl chains, and hydrophilic residues (AF) with trimethylammonium groups in the side chains. This amphiphilic structure induces the formation of distinct nanometer-scale phase-separated morphologies. For instance, transmission electron microscopy has revealed the presence of spherical hydrophilic domains of approximately 1.5 nm in diameter [19]. Small-angle X-ray scattering (SAXS) experiments have claimed that the average inter-cluster distance is approximately 8 nm based on the observation of a peak at ~0.8 nm^−1^. These observations imply the existence of a mesoscale structural organization. Subsequent investigation by Yoshida et al. employed wide-angle X-ray scattering (WAXS) [20] with high-intensity synchrotron radiation to delve deeper into the microscopic structures. The pair distribution functions were obtained at multiple water uptakes, and the hydration structures were discussed.

Despite the extensive efforts and the invaluable experimental data, interpreting the peak positions and variations observed in WAXS and SAXS remains nontrivial. Furthermore, the development of a consistent molecular model remains a significant challenge. To the best of our knowledge, despite a few seminal computational studies [21,22], a comprehensive structural comparison with the experiments, including WAXS and SAXS, has yet to be established. Consequently, due in part to the lack of reliable molecular models, the microscopic mechanism of water transport within AEMs remains to be fully elucidated.

In this study, to construct realistic AEM models and address microscopic mechanisms of water transport, all-atom models of QPAF-4 at four water uptakes were constructed, and molecular dynamics (MD) simulations were performed. The modeled systems spanned approximately 26 nm, enabling direct comparison with SAXS experiments. The scattering patterns calculated in this study were found to be consistent with the experimental SAXS spectra. Furthermore, the pair distribution functions obtained from simulations agreed well with those obtained from WAXS experiments. Therefore, these results demonstrate that the obtained structures reproduced both the mesoscale morphology and microscale molecular structure of the membrane. Beyond confirming consistency with the experiments, we investigated the factors contributing to the peaks and the variations observed in the WAXS and SAXS profiles. The analysis led us to hypothesize that the increase in water uptake enhances the aggregation of fluorinated alkyl chains. We proceed to a discussion of hydration-dependent water dynamics: the persistent subdiffusion of water molecules under conditions of low water uptake.

## 2. Model and Methods

### 2.1. Outline of Models

We constructed the QPAF-4 membrane systems in accordance with experimental data. We constructed a series of random copolymers with an overall PAF:AF ratio of 1:0.53. This composition reproduces the experimentally reported ion-exchange capacity of 1.5 meq/g [18] and matches that of the sample used by Yoshida et al. [20]. The length of each chain was set to 23 residues, which yielded a number-average molecular weight of approximately 10 kDa in accord with a typical experimental value [18]. In the simulation cell, 963 QPAF-4 chains were included to ensure a box length of ~25–27 nm, thereby ensuring adequate accommodation of the ≈8 nm water cluster spacing that was claimed by the SAXS experiments. Each of the 963 QPAF-4 chains had a unique sequence. In order to neutralize the system charge, 15,326 methyl sulfate ions were added as counterions, which were selected in accordance with the WAXS experiment [20]. Water molecules were introduced to reproduce water uptakes corresponding to the experimental relative humidity (30%RH, 50%RH, 70%RH, and 90%RH). In particular, the number of water molecules in the system was $N_{water}=$ 27,908, 46,327, 94,887, and 195,356, respectively, for 30% RH, 50% RH, 70% RH, and 90% RH.

The water uptakes, denoted by *λ*, are defined as the number of water molecules per trimethylammonium group:

(1) $$\lambda =\frac{N_{water}}{N_{TMA}}\;$$

where $N_{TMA}$ is the total number of trimethylammonium groups in the system. For the four aforementioned systems, the water uptakes are λ= 1.82, 3.02, 6.19, and 12.8. These systems are hereafter referred to as λ= 2, 3, 6, and 13. The total number of atoms, including dummy sites, ranged from approximately 1.4 to 2.1 million.

### 2.2. Molecular Dynamics Simulation Details

We used the OPLS-AA force field [23] with fixed charges that allow for long and large-scale simulations. Bonded and Lennard-Jones parameters for QPAF-4 were obtained from the LigParGen server [24,25]. Partial charges were assigned according to a protocol used in a previous study [26,27]. In this protocol, quantum chemical calculations were performed on the PAF and AF trimers. Structures were optimized at the B3LYP/6-31G(d) level using the Opt scheme, and electrostatic potentials were subsequently fitted using the ChelpG scheme at the B3LYP/6-311G(d,p) level with the Gaussian09 to derive partial charges. As the polymer is cationic, diffuse functions were not added to the basis set, and the functional used is also consistent with the literature [28]. The parameters for methyl sulfate ions, including partial charges, were obtained from the LigParGen server [24,25,29], and TIP4P/2005 was employed as the water model [30].

The initial structures were formed by random packing without atomic overlap using packmol [31]. The initial density was ≈0.072 g/cm^3^. Subsequently, the systems underwent a series of compression and annealing steps, following the protocol previously reported for Nafion membranes [32,33,34]. After this procedure, 5 ns NPT simulations were performed for an initial equilibration. We then performed NPT simulations for 300 ns to 500 ns, depending on water uptakes, as the production run. The final 200 ns of each simulation were used in analysis.

All production runs were performed using GROMACS 2022.6 [35]. The bonds involving hydrogen atoms were constrained by the LINCS algorithm [36], and a time step of 1 fs was employed. The long-range electrostatic interaction was treated with the particle mesh Ewald method [37], whereas a short-range cutoff of 1.2 nm was employed. The Bussi thermostat [38] was utilized to maintain the temperature at 333 K, and the Bernetti–Bussi barostat [39] was employed to control the pressure at 1 atm in accordance with the conditions of the WAXS experiments [20].

### 2.3. X-Ray Scattering Analysis

Elastic coherent scattering intensity was computed to compare SAXS, which typically probes the low scattering vector regime (Q≲1  nm^−1^), and WAXS, which typically probes the high scattering vector regime (Q≳1  nm^−1^). In the calculations, we used partial structure factor [40] obtained from MD calculations as follows. Letting $c_{\alpha }$ and $f_{\alpha }\left(Q\right)$ denote the molar fraction and atomic scattering factor of α-th element, respectively, the relative scattering intensity $I_{relative}\left(Q\right)$ was calculated using the following equation:

(2) $$I_{relative}\left(Q\right)=\langle f^{2}\left(Q\right)\rangle \;S\left(Q\right)\;$$

where

(3) $$\langle f^{2}\left(Q\right)\rangle =\sum_{\alpha =1}^{N_{elem}}c_{\alpha }f_{\alpha }^{2}\left(Q\right)\;$$

and $S\left(Q\right)$ is the (total) structure factor. Here, $N_{\mathrm{elem}}$ denotes the number of elements or distinct kinds of atoms. The structure factor can be obtained from the Faber–Ziman type partial structure factor $S_{\alpha \beta }\left(Q\right)$. Letting ρ and $g_{\alpha \beta }\left(r\right)$ be the number density of all atoms and the partial radial distribution function between elements α and β, respectively, the partial structure factors can be evaluated by

(4) $$S_{\alpha \beta }\left(Q\right)-1\equiv \rho \int_{0}^{\infty }\left(g_{\alpha \beta }\left(r\right)-1\right)\frac{\sin Qr}{Qr}4\pi r^{2}dr\;$$

Here, $g_{\alpha \beta }\left(r\right)$ can be readily obtained from the MD calculation. The total structure factor $S\left(Q\right)$ can be obtained as weighted sum of $S_{\alpha \beta }\left(Q\right)-1$:

(5) $$S\left(Q\right)-1=\sum_{\alpha =1}^{N_{elem}}\sum_{\beta =\alpha }^{N_{elem}}w_{\alpha \beta }\left(Q\right)\;\left(S_{\alpha \beta }\left(Q\right)-1\right)\;\;\;$$

where letting $\delta_{\alpha \beta }$ be the Kronecker delta, the weight $w_{\alpha \beta }\left(Q\right)$ is given by

(6) $$w_{\alpha \beta }\equiv \left(2-\delta_{\alpha \beta }\right)\;\;\frac{c_{\alpha }c_{\beta }f_{\alpha }\left(Q\right)f_{\beta }\left(Q\right)}{\langle f^{2}\left(Q\right)\rangle }$$

Therefore, $S\left(Q\right)$ can be seen as a weighted sum of Fourier-transformed radial distribution function. Given Equation (5), the contribution to $S\left(Q\right)-1$ from each pair was evaluated by the weighted partial structure factor:

(7) $$S_{\alpha \beta }^{weighted}\left(Q\right)\equiv w_{\alpha \beta }\left(S_{\alpha \beta }\left(Q\right)-1\right)$$

In order to ensure numerical stability, the Lorch window function $M\left(r;R\right)=sinc\pi r/R$ was employed to calculate $S_{\alpha \beta }\left(Q\right)$, and the integration was terminated at $R=\frac{L}{2}\approx 13$ nm:

(8) $$S_{\alpha \beta }\left(Q\right)-1\equiv \rho \int_{0}^{R}\left(g_{\alpha \beta }\left(r\right)-1\right)M\left(r\right)\frac{\sin Qr}{Qr}4\pi r^{2}\;dr$$

The lowest wavenumber was $Q_{min\;}=\frac{2\pi }{R}\approx 0.48$ nm^−1^. The highest wavenumber was set to $Q_{max\;}=$ 220 nm^−1^ in accordance with the WAXS experiment [20]. The resolution of Q was set to ΔQ=0.1 nm^−1^. The atomic scattering factors for each element were obtained from the literature [41]. The calculations were performed using in-house code.

### 2.4. Analysis of Pair Distribution Function

To make a comparison with WAXS experimental results, we calculated the pair distribution function $G\left(r\right)\equiv 4\pi \rho r\left[g\left(r\right)-1\right]$. In accordance with the WAXS experiment [20], we evaluated the pair distribution function via inverse Fourier transformation of the modified structure factor

(9) $$\begin{array}{lll} S^{\prime }\left(Q\right)-1 & \equiv & \frac{\langle f^{2}\left(Q\right)\rangle }{{\langle f\left(Q\right)\rangle }^{2}}\left(S\left(Q\right)-1\right)\\ & = & \sum_{\alpha =1}^{N_{\mathrm{elem}}}\sum_{\beta =\alpha }^{N_{\mathrm{elem}}}w_{\alpha \beta }^{\prime }\left(Q\right)\;\left(S_{\alpha \beta }\left(Q\right)-1\right) \end{array}$$

where

(10) $$w_{\alpha \beta }^{\prime }\left(Q\right)=\left(2-\delta_{\alpha \beta }\right)\;\;\frac{c_{\alpha }c_{\beta }f_{\alpha }\left(Q\right)f_{\beta }\left(Q\right)}{{\langle f\left(Q\right)\rangle }^{2}}\;$$

whereas one school [42] advocates the use of $S\left(Q\right)$ in lieu of $S^{\prime }\left(Q\right)$. In accordance with the experimental analysis [20], the Lorch window function $M\left(Q;Q_{max}\right)=\mathrm{sinc}\pi Q/Q_{max}$ was employed, and $G\left(r\right)$ was evaluated as follows:

(11) $$G_{MD,FFT}\left(r\right)\equiv \frac{\pi }{2}\;\int_{0}^{Q_{max}\;}Q\left.\left(S^{\prime }\left(Q\right)-1\right.\right)M\left(Q\right)\sin QrdQ$$

where $Q_{max}\approx 220$ nm^−1^ in accordance with the experiments. The resolution in real space is $\frac{2\pi }{Q_{max}}\approx 0.03$ nm, which is coarser than the resolution achievable by all-atom MD calculations. Consequently, whereas $G_{MD,FFT}\left(r\right)$ is directly pertinent to experimental observation, it does not fully preserve the resolution of the all-atom MD simulations.

To take full advantage of MD simulation, we defined an atomic number weighted average pair distribution function as follows. Given the approximation of $\frac{f_{\alpha }\left(Q\right)}{f_{\beta }\left(Q\right)}\approx \;\frac{Z_{\alpha }}{Z_{\beta }}$, the weight $w_{\alpha \beta }^{\prime }\left(Q\right)$ can be approximated as follows:

(12) $$w_{\alpha \beta }^{\prime }\left(Q\right)\approx \left(2-\delta_{\alpha \beta }\right)\;\;\frac{c_{\alpha }c_{\beta }Z_{\alpha }Z_{\beta }}{{\langle Z\rangle }^{2}}\equiv w_{\alpha \beta }^{\prime \prime }\;$$

where $\langle Z\rangle =\sum_{\alpha =1}^{N_{elem}}c_{\alpha }Z_{\alpha }$ is the average of atomic numbers. The atomic number weighted average pair distribution function can then be defined as follows:

(13) $$G_{MD}\left(r\right)=4\pi \rho r\left[\sum_{\alpha =1}^{N_{elem}}\sum_{\beta =\alpha }^{N_{elem}}w_{\alpha \beta }^{\prime \prime }\left(g_{\alpha \beta }\left(r\right)-1\right)\right]$$

It can be easily demonstrated that $G_{MD,FFT}\left(r\right)\to \;G_{MD}\left(r\right)$ for $Q_{max}\to \infty ,$ under the assumption that $w_{\alpha \beta }^{\prime }\left(Q\right)=w_{\alpha \beta }^{\prime \prime }$, as $w_{\alpha \beta }^{\prime \prime }$ is independent of Q. The contribution of each component was defined as follows:

(14) $$G_{\alpha \beta }^{weighted}\left(r\right)=4\pi \rho r{\;w}_{\alpha \beta }^{\prime \prime }\;\left(g_{\alpha \beta }\left(r\right)-1\right)$$

This quantity is instrumental in interpreting the peaks and variations in $G\left(r\right)$. This approach enables a direct comparison between the MD results and the experimental ones, whereas preserving the full atomistic resolution through MD simulations.

## 3. Results and Discussion

### 3.1. Equilibration and Mass Density

Because polymeric systems typically require long timescales to reach equilibrium, achieving complete equilibration within the timescale of MD simulations remains a significant challenge. In this study, simulations were performed from 300 ns to 500 ns and truncated after the mass density converged.

**Figure 2:**
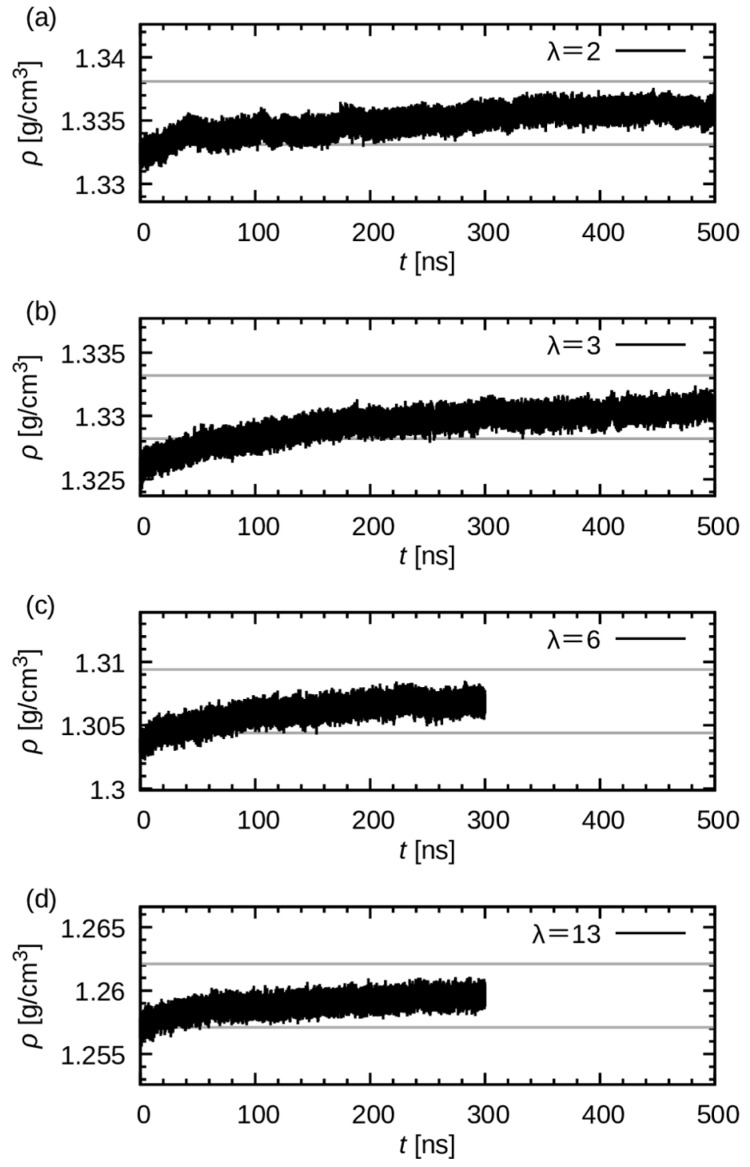
Time evolution of mass density of the system. The panels (**a**), (**b**), (**c**), and (**d**) represent the results of λ = 2, 3, 6, and 13, respectively. The gray lines are drawn at $\rho_{final}\pm 0.0025$ g/cm^3^ to verify the convergence.

Figure 2 shows the time evolution of mass density in the production runs. The time required for density convergence is longer in systems with lower water uptake. For systems with higher water uptake, the density tends to stabilize relatively early from the beginning. This observation indicates that the presence of water molecules has a plasticizing effect on the polymer matrix, thereby accelerating structural relaxation.

Given the finding that relaxation was slower in the two systems with smaller water uptakes, the simulation time was extended to 500 ns. Consequently, at the final 200 ns, the change in the density nearly ceased, signifying that adequate convergence was achieved. Accordingly, the final 200 ns were considered in the following analyses.

**Figure 3:**
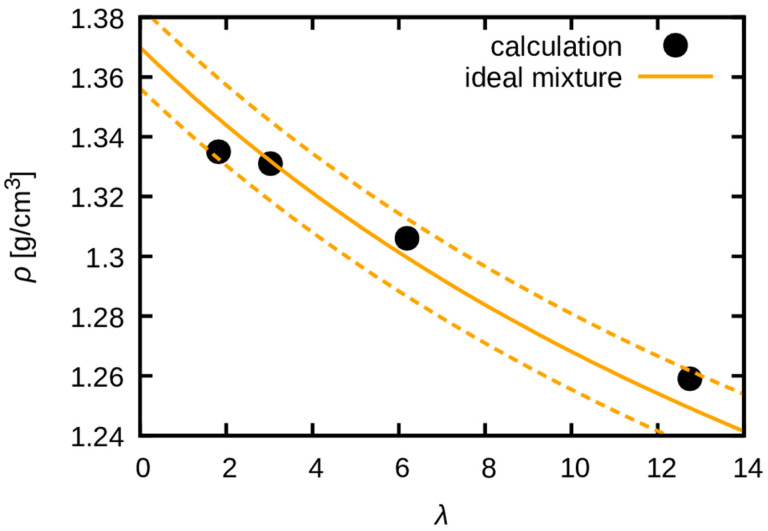
Calculated density as a function of water uptake, λ, is represented by black circles in the graph. The solid orange line represents the ideal mixture. The orange dashed lines represent the 1% deviation from the ideal mixture.

A prior experimental study reported difficulty in quantifying the extent of swelling due to the complexity in measuring the volume of a hydrated polymer [22]. Therefore, the volume swelling behavior was estimated by calculating the dependence of mass density on water uptake (Figure 3). The results indicate that the density is consistent with the ideal mixing of polymer and water. Consequently, the swollen volume can be predicted if the water uptake is known. This finding validates the practice of using an ideal mixture to estimate swollen membranes’ density, which was needed for the analysis of the WAXS experiment [20].

### 3.2. Structures and Their Comparison with X-Ray Experiments

**Figure 4:**
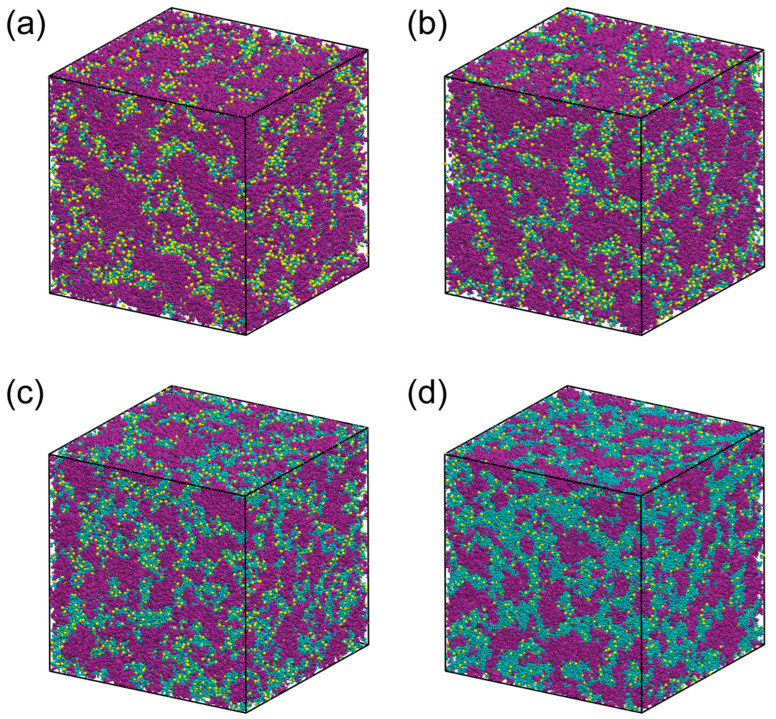
Visual representations of the electrolyte following equilibration. (**a**), (**b**), (**c**), and (**d**) represent λ = 2, 3, 6, and 13, respectively. Atoms in polymers are represented in purple. Water molecules are represented in cyan. The sulfur atoms in methyl sulfate ions are represented by yellow spheres.

Figure 4 presents representative snapshots of the QPAF-4 systems, which clearly demonstrate complex microphase separation between the water and polymer phases. The morphology of the system changes in response to water uptake. The simulated cell has a side length of approximately 25 nm to 27 nm, and several water clusters of a few nm in size are formed. The length of the simulation cell is large enough to accommodate the ≈8 nm spacing reported in the SAXS experimental study. Consequently, a comparison can be made between the present simulation and the SAXS experiment. Furthermore, the utilization of all-atom models enables the comparison of the pair distribution between WAXS and the present calculation. In this section, a comprehensive comparison is made with experimental structural observations.

#### 3.2.1. Comparison with SAXS

**Figure 5:**
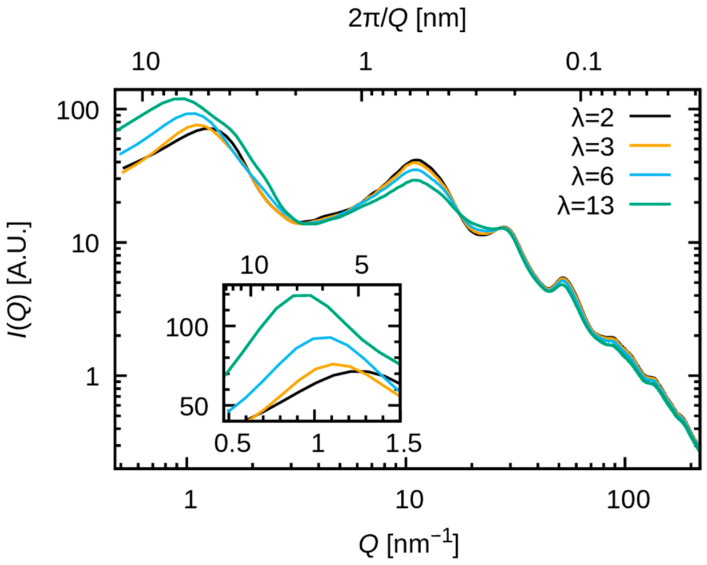
Calculated relative intensity is plotted as a function of Q. The inset shows the same plot with a different scale and range. The black, orange, cyan, and green lines correspond to λ = 2, 3, 6 and 13, respectively.

Figure 5 illustrates the relative intensity of X-ray scattering, as calculated by Equation (2). The minimum wavenumber is expressed as $Q_{min\;}=2\pi /(L/2)$, where L denotes the cell size. In the present study, with L≈ 25 nm to 27 nm $Q_{min\;}\approx 0.48$ nm^−1^ was obtained. The peaks found from Q≈0.8 nm^−1^ to 1.1 nm^−1^ are in good agreement with the findings of experimental studies (see, e.g., Figure 4b in ref. [18]). The experimental results also exhibit a shift in the peak toward smaller Q values with increasing water uptake. These observations indicate that the simulated system reproduced mesoscale structures (6–10 nm range) associated with hydrophilic/hydrophobic segregation.

**Figure 6:**
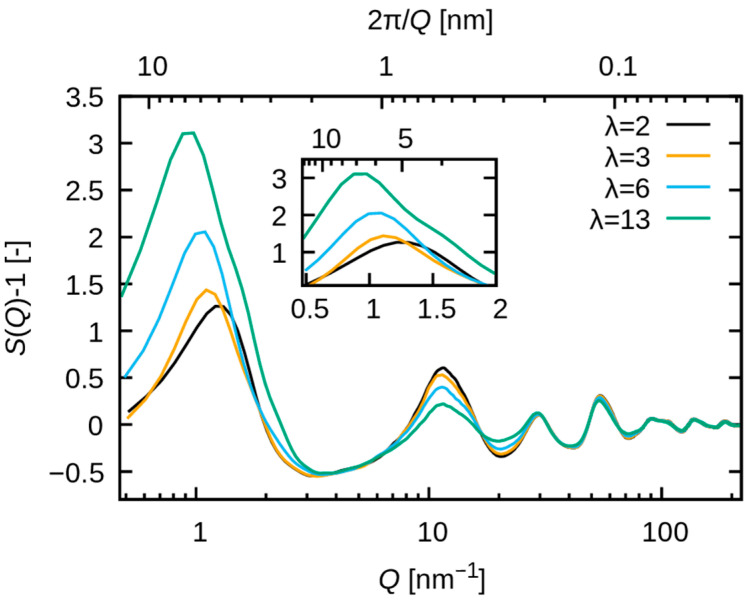
Calculated S(Q)−1 is plotted as a function of Q. The black, orange, cyan, and green lines correspond to λ = 2, 3, 6 and 13, respectively.

**Figure 7:**
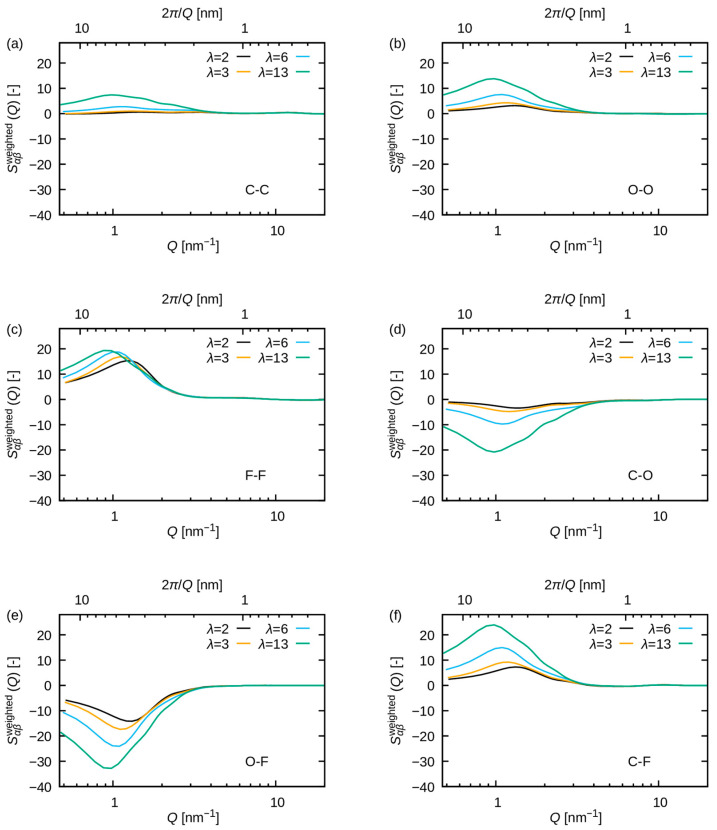
The pair-wise contribution $S_{\alpha \beta }^{weighted}\left(Q\right)$ for (**a**) C-C, (**b**) O-O, (**c**) F-F, (**d**) C-O, (**e**) O-F, and (**f**) C-F. The black, orange, cyan, and green lines correspond to λ = 2, 3, 6 and 13, respectively.

Figure 6 illustrates the structure factors $S\left(Q\right)-1,$ which demonstrate a strong correlation with the intensity $I\left(Q\right)$. Consequently, the observed variation in $I\left(Q\right)$ is attributed to the variation in $S\left(Q\right)$, which stems from pair distribution functions. Figure 7 shows $S_{\alpha \beta }^{weighted}(Q)$ with a large contribution to $S\left(Q\right)$ at Q≈1 nm^−1^. The peak of S(Q) observed at Q≈1 nm^−1^ is comprised of multiple contributions, including not only the O-O pair but also significant contributions from the F-F and C-F pairs. Consequently, the peak is attributable to the periodicity of the polymer matrix around 4 to 8 nm, in conjunction with that of water molecules. In contrast, O-F and C-O exhibit negative contributions, suggesting that within the phase-separated structure, the formation of periodicity spanning 4–8 nm is precluded for these pairs.

The water uptake dependence is more pronounced for F-F pairs than for O-O pairs [see Figure 7b,c]. A low wavenumber shift of the peak with increasing water uptake is observed for both pairs. These results indicate that the period of correlation for both polymer matrix and water clusters increases with increasing water uptake. The results also indicate that the increase in the peak at Q≈1 nm^−1^ for increasing water uptake is attributed to both F-F pairs and O-O pairs. The increase in the F-F pairs peak can be attributed to the change in $S_{\alpha \beta }\left(Q\right)$ (see Figure S1), whereas that of the O-O pairs results from the increase in $w_{\alpha \beta }$ due to the rising molar fraction of oxygen atoms. This observation indicates that the addition of water may promote the aggregation of fluorinated alkyl chains. The experimental validation of this hypothesis, for example using isotopic substitution and neutron scattering experiments, will be interesting in future studies.

**Figure 8:**
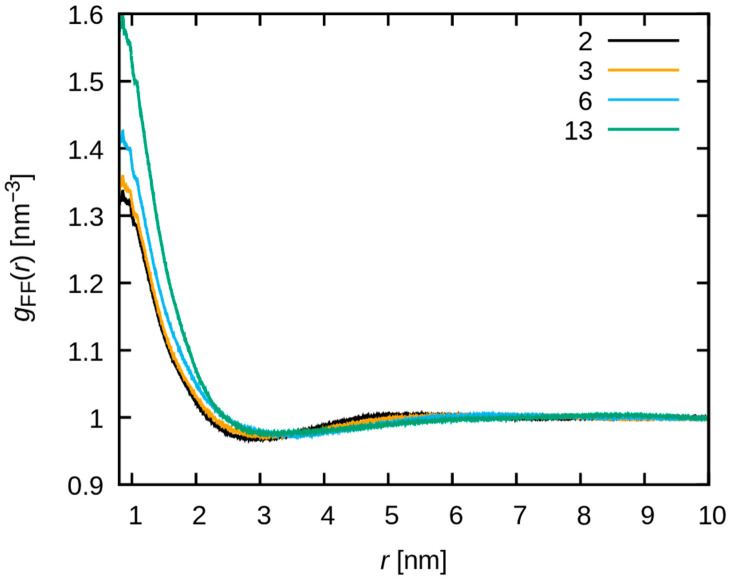
The F-F radial distribution function $g_{FF}(r)$ is plotted. The black, orange, cyan, and green lines correspond to λ = 2, 3, 6 and 13, respectively.

**Figure 9:**
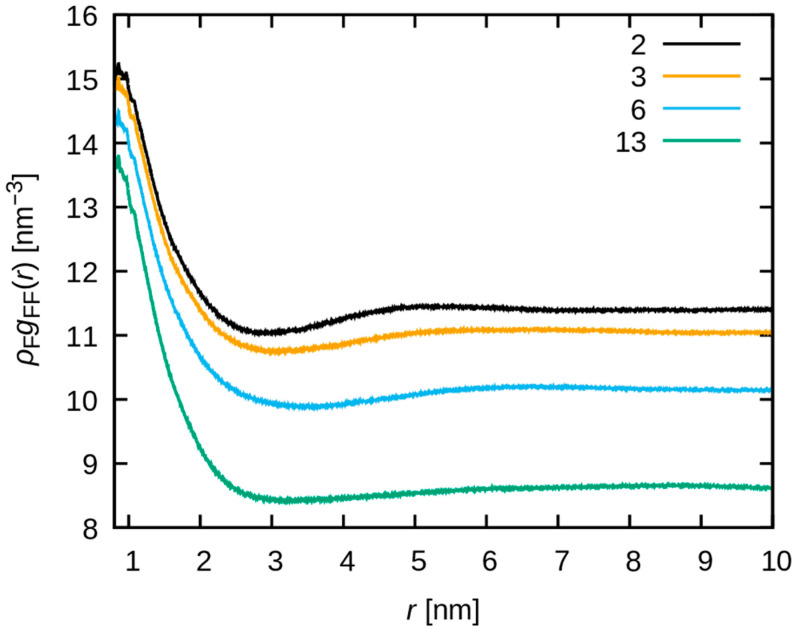
The scaled F-F radial distribution function $\rho_{F}g_{FF}(r)$ is plotted. The black, orange, cyan, and green lines correspond to λ = 2, 3, 6 and 13, respectively.

To further investigate the aggregation behavior of F-F pairs, $g_{FF}\left(r\right)$ and $\rho_{F}g_{FF}\left(r\right)$ are shown for r≥0.8 nm to exclude intra-molecular correlation in Figure 8 and Figure 9, respectively. The $g_{FF}\left(r\right)$ value around 1 nm increases at the water uptake increases (Figure 8). Because of the dilution of fluorine, $\rho_{F}g_{FF}\left(r\right)$ slightly decreases around 1 nm at increasing water uptake, but the largest change is observed at the highest water uptake (Figure 9). This also suggests the F-F aggregation at increasing water uptake, as represented in the real space. In contrast, the C-C and O-O pairs exhibit a distinct behavior from F-F pairs (see Figure S2–S5 in SI).

The fluorine aggregation may originate from differences in miscibility. Surface tension measurements [43] indicated that perfluoroalkanes are more immiscible with water than alkanes. With increasing water uptake, it is possible that the perfluoroalkane segments aggregate further to minimize the interfaces with water and that the hydrocarbon segments serve as a bridge between the perfluoroalkane aggregates and water domains.

#### 3.2.2. Comparison with WAXS

**Figure 10:**
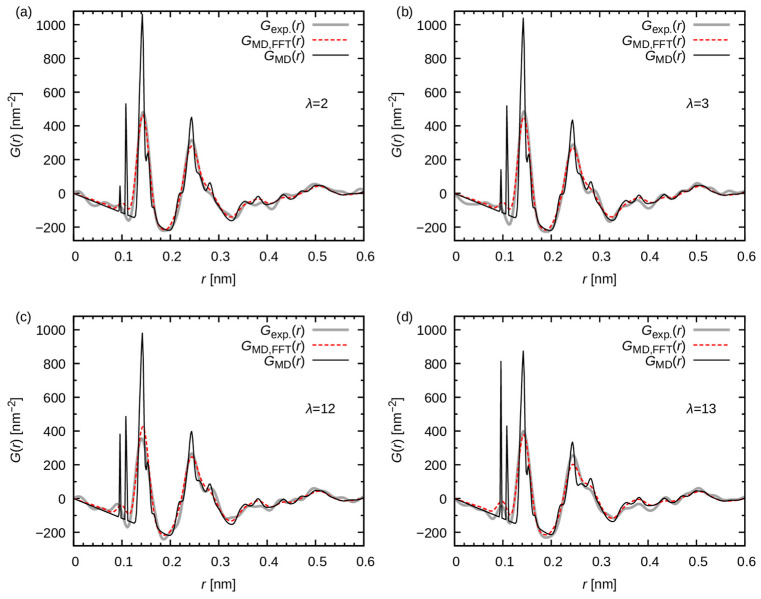
Pair distribution function as a function of r. The gray thick, red dashed, and black solid lines represent $G_{exp}(r)$, $G_{MD,FFT}(r)$, and $G_{MD}(r)$, respectively. The panels (**a**), (**b**), (**c**), and (**d**) correspond to the data at λ = 2, 3, 6, and 13, respectively.

Figure 10 shows the pair distribution functions, which are used to compare the experiments and present calculations. The shape of $G_{MD}(r)$ (black solid lines) is sharper than the experimentally obtained curve, and narrow peaks emerge around 0.1 nm. The presence of these narrow peaks can be attributed to the hydrogen atoms. Conversely, $G_{exp}(r)$ (gray thick lines) exhibits only minor broad peaks around 0.1 nm. These disparities can be attributed to the limitation in the spatial resolution of the experimental apparatus, as described below.

To compare the pair distribution function under the experimental condition, $G_{MD,FFT}(r)$ was also calculated (red dashed lines in Figure 10). As previously mentioned in Section 2.4, $G_{MD,FFT}(r)$ was obtained by the inverse Fourier transformation of the calculated structure factor. In this inverse Fourier transformation, the experimental limitation of Q (approximately 220 nm^−1^) was explicitly considered in conjunction with the Lorch window function.

As demonstrated in Figure 10, the resulting $G_{MD,FFT}(r)$ closely aligns with the experimental profile $G_{exp}(r)$, thereby substantiating the consistency between the WAXS experiments and the present calculations. The discrepancy between $G_{MD,FFT}(r)$ and $G_{MD}(r)$ underscores the loss of high-wavenumber components in the experiment, attributable to instrumental limitations. Consequently, MD simulations should be considered indispensable for comprehending and interpreting the experimental profile.

**Figure 11:**
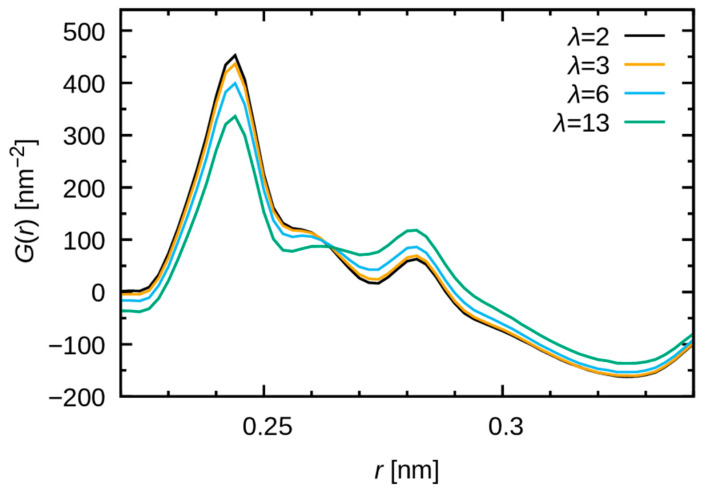
Peaks of $G_{MD}(r)$ in the vicinity of 0.28 nm. Black, yellow, cyan, and green indicate water uptake at λ = 2, 3, 6, and 13, respectively.

**Figure 12:**
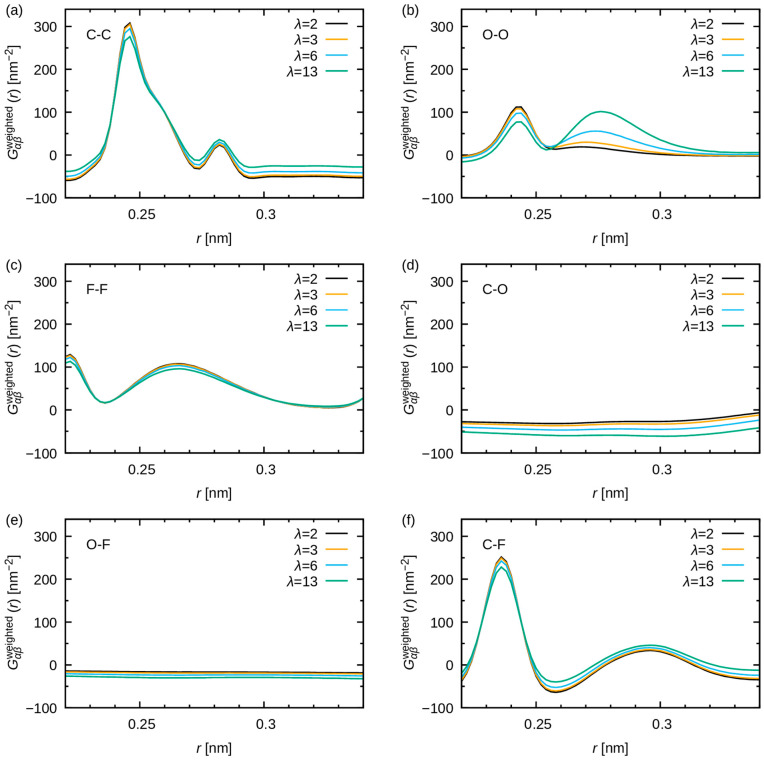
Representative components $G_{\alpha \beta }^{weighted}\left(r\right)$ of $G_{MD}(r)$ in the vicinity of 0.28 nm are plotted. Panel (a), (b), (c), (d), (e), (f) illustrate the contributions from the C-C, O-O, F-F, C-O, O-F, and C-F pairs, respectively. The colors black, yellow, cyan, and green are used to indicate water uptake at λ = 2, 3, 6 and 13, respectively.

Figure 11 highlights the λ dependence around 0.28 nm. The height of peak near 0.28 nm increases with increasing water uptake, consistent with the observations from the WAXS study [20]. Figure 12 provides a decomposition of the contributions made by atomic pairs. The peak produced by the O-O pairs is broad, but the height increases significantly around 0.28 nm as the water uptake increases. This finding supports the conclusions of a preceding study, which attributed the 0.28 nm feature to water–water correlations within the hydrophilic domains [20]. Additionally, marginal peaks in O-O pairs at 0.28 nm can be observed, in cases of low water uptake (λ = 2 and 3). Consequently, a negligible number of water molecules form hydrogen bonds with other water molecules within the cluster. Most water molecules are utilized in the hydration shells of the polymer and methyl sulfate ions.

The present results also indicate that a delicate discussion of the peak shape is needed, as the overall shapes around 0.28 nm are ascribed to multiple overlapping profiles. For instance, a narrow peak originating from C-C pairs emerges at 0.282 nm, contributing to a minor peak in the overall pair distribution function (Figure 11). This analysis illustrates that because of the existence of multiple peaks in similar locations, MD simulations are imperative for the assignment and discussion of peak shapes and variations. It is important to note that the changes observed in the C-C, F-F, C-O, and O-F, C-F pairs in Figure 12 are derived from the variations in the concentration of each atom rather than from those in the structure.

In summary, the molecular structures obtained from MD simulations are highly consistent with the SAXS and WAXS experiments, indicating that the present calculations are realistic. Furthermore, the reliable structural descriptions are valuable for interpreting experimental data and offer new and deeper insights into the system.

### 3.3. Dynamics of Water Molecules

**Figure 13:**
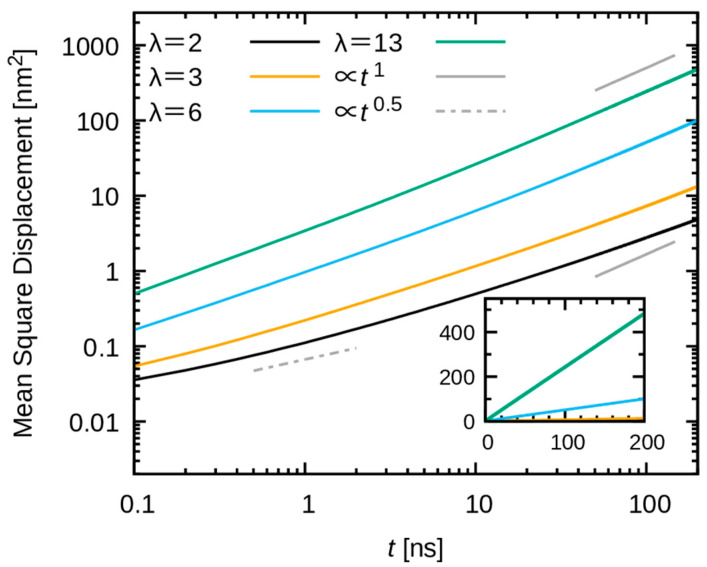
Mean square displacement (MSD) of water molecules as a function of time. Black, yellow, cyan, and green indicate water uptakes λ = 2, 3, 6 and 13, respectively. The inset shows the linear plot with a different range, where the black and orange lines are almost invisible because of too-small increasing rates.

As the structural fidelity of the model was confirmed in the preceding sections, the subsequent investigation focused on the dynamics of water. Figure 13 shows the mean square displacement (MSD) of water molecules. The MSD increases more rapidly as a function of time as the water uptake increases. For the low water uptakes (λ = 2 and 3), the increase in MSD is significantly slower (see the inset in Figure 13). In addition to the rate of increase in MSD, a substantial impact of water uptake on the behavior of the MSD is evident. At elevated water uptakes (λ = 6 and 13), the MSD exhibited a nearly linear increase with time at ≈200 ns, indicating an approach to diffusive behavior. In contrast, for systems with low water uptakes (i.e., λ = 2 and 3), the MSD is clearly nonlinear as a function of time. The subdiffusive behavior persists even at ≈200 ns. These slow and distinct behaviors manifest at low water uptake, and a potential explanation for these behaviors lies in the confinement of water molecules within a narrow space, along with the strong interaction exerted by the polymers and methyl sulfate ions.

**Figure 14:**
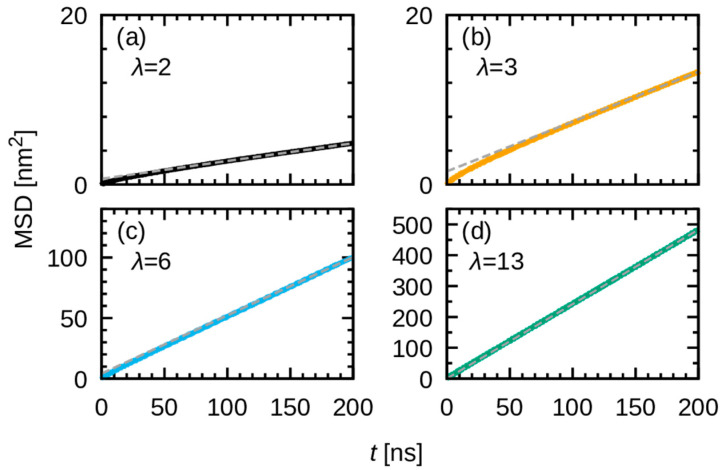
Fitting of linear function to the final 20 ns of MSD. Panels (**a**), (**b**), (**c**), and (**d**) represent λ = 2, 3, 6, and 13, respectively.

**Figure 15:**
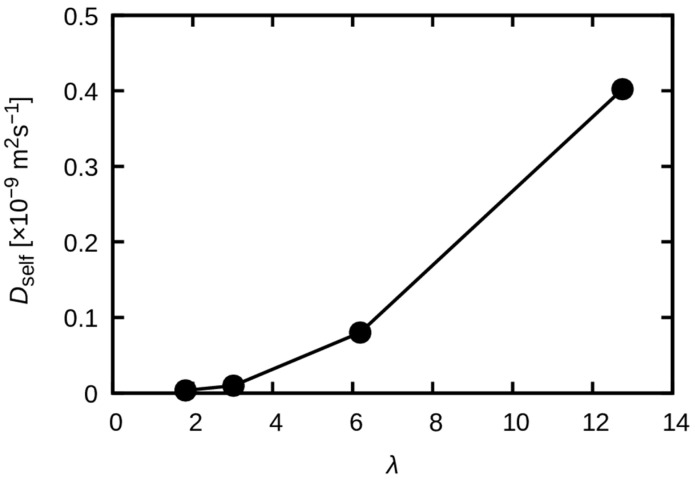
Apparent diffusion coefficient of water molecules represented as a function of water uptake.

Even though the diffusion coefficient might not be rigorously defined due to the nonlinear behavior, even at 200 ns for λ = 2 and 3, an apparent diffusion coefficient was estimated from a linear fit to the final 20 ns (180 ns to 200 ns) of the MSD curves (Figure 14). The water uptake dependence thus obtained is shown in Figure 15. The findings indicate that the diffusion coefficient does not demonstrate a monotonous linear relationship with *λ*; rather, it exhibits a nonlinear behavior that increases rapidly above a certain threshold value. However, a conclusive interpretation necessitates the execution of longer replicated simulations, which require substantial computational resources and will be addressed in future work.

## 4. Conclusions

In this study, we conducted large-scale all-atom MD simulations on the anion-exchange membrane QPAF-4. The simulated systems consisted of approximately 1.4 to 2.1 million atoms under various water uptake conditions. These large-scale simulations were carried out up to 500 ns, depending on the system.

A thorough comparison was conducted between the structure obtained from MD simulations and SAXS and WAXS experiments. The structure factors and pair distribution functions obtained from the simulations agreed with the experimental results excellently. Hence, the structural integrity of the molecular structures is validated not only on the microscale covered by WAXS but also on the mesoscale (ranging from a few nm to 10 nm) covered by SAXS. The use of the all-atom models facilitated a direct comparison of pair distribution functions, which are not accessible to coarse-grained models. Moreover, the utilization of gigantic systems enabled the coverage of the SAXS-relevant wavevector range (Q ≈ 0.5–1.1 nm^−1^), which is ordinarily inaccessible to small systems.

By analyzing the weighted partial structure factor $S_{\alpha \beta }^{weighted}(Q)$, the peak observed in SAXS at  Q≈1 nm^−1^ was ascribed not only to O-O pairs but also to F-F and C-F pairs. The increase in the F-F pairs peak can be attributed to the change in $S_{\alpha \beta }(Q)$, whereas that of the O-O pairs results from the increase in the weight $w_{\alpha \beta }$ arising from the increasing molar fraction of oxygen atoms. This suggests that the incorporation of water promotes the aggregation of fluorinated alkyl chains, thereby augmenting the periodicity of F-F pairs within the range of 4 to 8 nm. This hydration-induced aggregation is further supported by the observed increase in the radial distribution function $g_{FF}\left(r\right)$ for F-F pairs around 1 nm with increasing water uptake. Such behavior may be ascribed to the differences in miscibility among hydrocarbons, perfluorocarbons, and water molecules.

We also analyzed the translational diffusion of water molecules. For high water uptake (*λ* = 6 and 13), the water molecules exhibited almost diffusive behavior on ~200 ns, whereas for low water uptakes (*λ* = 2 and 3), the MSD displayed persistent subdiffusvie behavior. This persistent subdiffusve behaviors exhibited by these systems may be attributed to the hydration structures and the confinements present within the complex nanospace.

This work demonstrates that all-atom MD simulations, when applied at sufficiently large scales, are capable of reproducing both SAXS and WAXS data. Furthermore, they provide molecular-level insights into the structure and transport in AEM materials. These findings provide a robust theoretical basis for the rational design of next-generation anion-exchange membranes.

## Data Availability

The datasets presented in this article are not readily available because the data are part of an ongoing study. Requests to access the datasets should be directed to the corresponding author.

## Supplementary Materials

The following supporting information can be downloaded at: https://www.mdpi.com/article/10.3390/membranes15090266/s1, Figure S1: The partial structure factors $S_{\alpha \beta }(Q)$; Figure S2: The C-C radial distribution function $g_{CC}\left(r\right)$; Figure S3: The scaled C-C radial distribution function $\rho_{C}g_{CC}\left(r\right)$; Figure S4: The O-O radial distribution function $g_{OO}\left(r\right)$;.Figure S5: The scaled O-O radial distribution function $\rho_{O}g_{OO}\left(r\right)$.
