# Supplementary material for: Large-Scale Molecular Dynamics of Anion-Exchange Membranes: Molecular Structure of QPAF-4 and Water Transport
Source: Membranes (Basel). 2025 Sep 2;15(9):266. doi: 10.3390/membranes15090266 (PMC12471824; doi:10.3390/membranes15090266)
Supplement: Supplementary file 1 [file membranes-15-00266-s001.zip › membranes-3784869-supplementary.pdf]

Supporting Information: Large-Scale Molecular Dynamics of Anion-Exchange Membranes: Molecular Structure of QPAF-4 and Water Transport

Tetsuro Nagai[1,*], Takumi Kawaida[2], and Koji Yoshida[1]

[1]*Department of Chemistry, Faculty of Science, Fukuoka University, 8-19-1 Nanakuma, Jonan-ku, Fukuoka 814-0180, Japan*

[2]*Graduate School of Science, Fukuoka University, 8-19-1 Nanakuma, Jonan-ku, Fukuoka 814-0180, Japan*

[*]Correspondence: tnagai@fukuoka-u.ac.jp

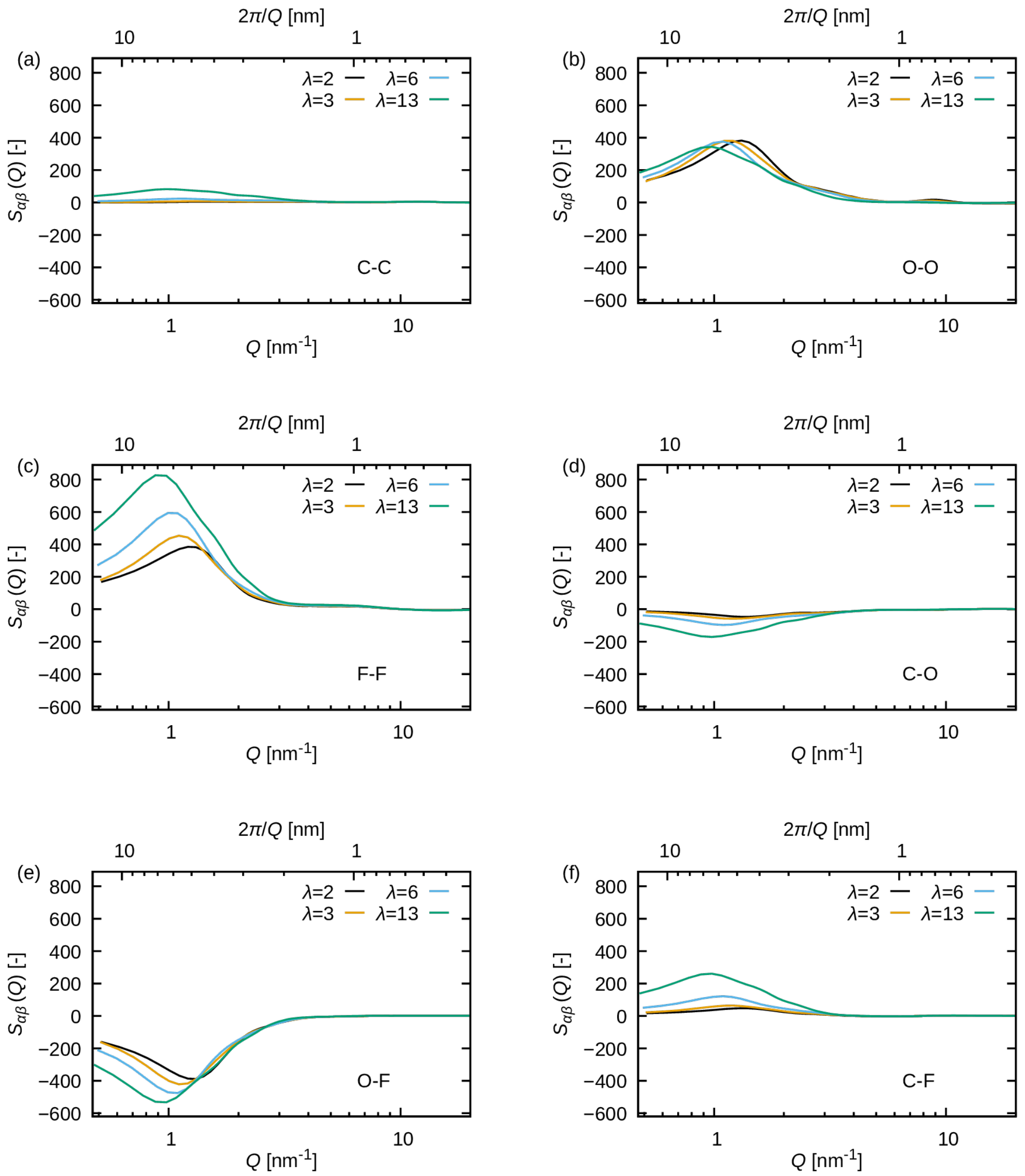

Fig. S1. The partial structure factor $S_{\alpha\beta}(Q)$ for (a) C-C, (b) O-O, (c) F-F, (d) C-O, (e) O-F, and (f) C-F. The black, orange, cyan, and green lines correspond to λ= 2, 3, 6 and 13, respectively.

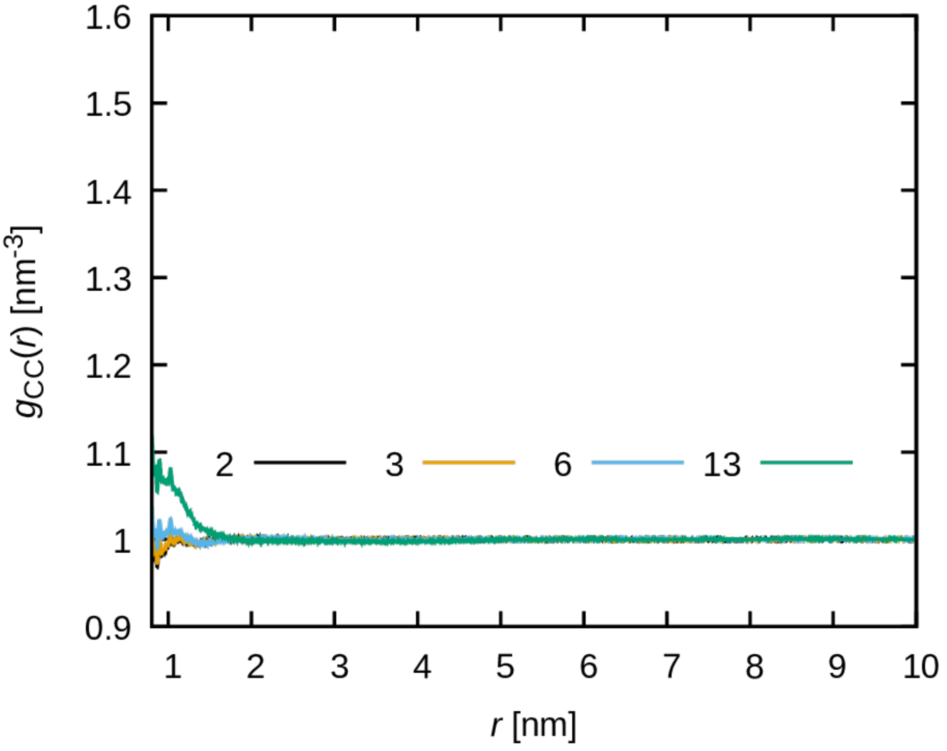

Fig. S2. The C-C radial distribution function $g_{CC}(r)$ is plotted. The black, orange, cyan, and green lines correspond to λ= 2, 3, 6 and 13, respectively.

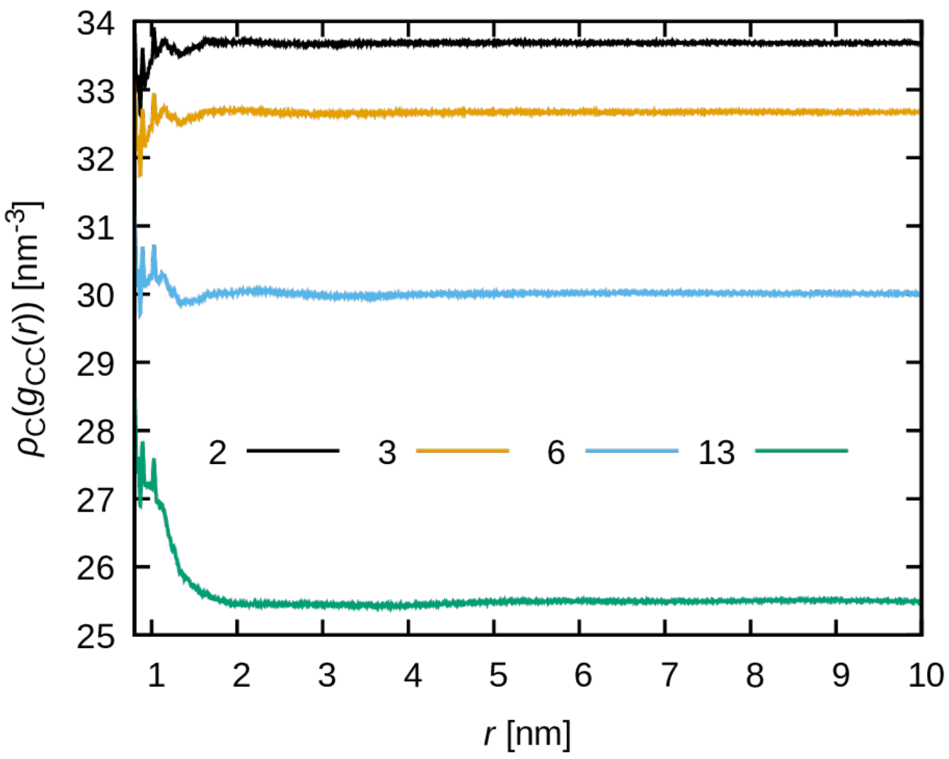

Fig. S3. The scaled C-C radial distribution function $\rho_C g_{CC}(r)$ is plotted. The black, orange, cyan, and green lines correspond to λ= 2, 3, 6 and 13, respectively.

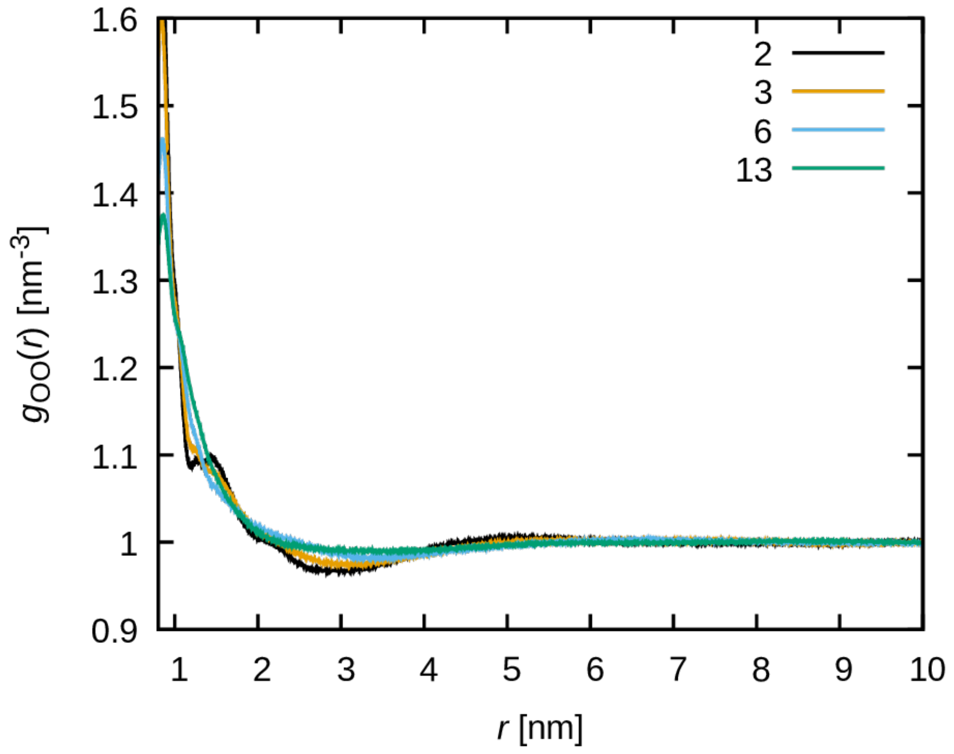

Fig. S4. The O-O radial distribution function $g_{\mathrm{OO}}(r)$ is plotted. The black, orange, cyan, and green lines correspond to λ= 2, 3, 6 and 13, respectively.

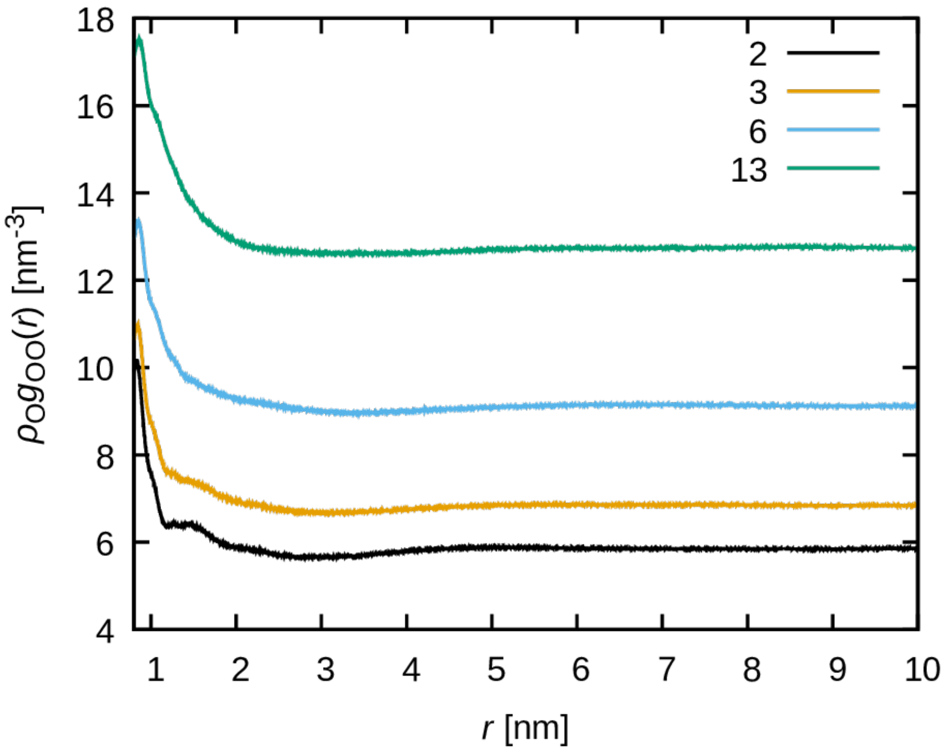

Fig. S5. The scaled O-O radial distribution function $\rho_O g_{OO}(r)$ is plotted. The black, orange, cyan, and green lines correspond to λ= 2, 3, 6 and 13, respectively.
